# Photobiomodulation at Defined Wavelengths Regulates Mitochondrial Membrane Potential and Redox Balance in Skin Fibroblasts

**DOI:** 10.1155/2023/7638223

**Published:** 2023-08-24

**Authors:** Vito Antonio Baldassarro, Giuseppe Alastra, Luca Lorenzini, Luciana Giardino, Laura Calzà

**Affiliations:** ^1^Department of Veterinary Medical Sciences, University of Bologna, Via Tolara di Sopra 50, Ozzano dell'Emilia (Bologna) 40064, Italy; ^2^Interdepartmental Centre for Industrial Research in Health Sciences and Technologies, University of Bologna, Via Tolara di Sopra 41/E, Ozzano dell'Emilia (Bologna) 40064, Italy; ^3^Department of Pharmacy and Biotechnology, University of Bologna, Via Belmeloro 6, Bologna 40126, Italy; ^4^IRET Fundation, Via Tolara di Sopra 41/E, Ozzano dell'Emilia (Bologna) 40064, Italy

## Abstract

Starting from the discovery of phototherapy in the beginning of the last century, photobiomodulation (PBM) has been defined in late 1960s and, since then, widely described in different *in vitro* models. Robust evidence indicates that the effect of light exposure on the oxidative state of the cells and on mitochondrial dynamics, suggesting a great therapeutic potential. The translational scale-up of PBM, however, has often given contrasting and confusing results, mainly due to light exposure protocols which fail to adequately control or define factors such as emitting device features, emitted light characteristics, exposure time, cell target, and readouts. In this *in vitro* study, we describe the effects of a strictly controlled light-emitting diode (LED)-based PBM protocol on human fibroblasts, one of the main cells involved in skin care, regeneration, and repair. We used six emitter probes at different wavelengths (440, 525, 645, 660, 780, and 900 nm) with the same irradiance value of 0.1 mW/cm^2^, evenly distributed over the entire surface of the cell culture well. The PBM was analyzed by three main readouts: (i) mitochondrial potential (MitoTracker Orange staining), (ii) reactive oxygen species (ROS) production (CellROX staining); and (iii) cell death (nuclear morphology). The assay was also implemented by cell-based high-content screening technology, further increasing the reliability of the data. Different exposure protocols were also tested (one, two, or three subsequent 20 s pulsed exposures at 24 hr intervals), and the 645 nm wavelength and single exposure chosen as the most efficient protocol based on the mitochondrial potential readout, further confirmed by mitochondrial fusion quantification. This protocol was then tested for its potential to prevent H_2_O_2_-induced oxidative stress, including modulation of the light wave frequency. Finally, we demonstrated that the controlled PBM induced by the LED light exposure generates a preconditioning stimulation of the mitochondrial potential, which protects the cell from oxidative stress damage.

## 1. Introduction

The beneficial effect of light exposure on living organisms has been anecdotally reported since ancient times. In 1903, the Nobel Prize in medicine and physiology was awarded to Ryberg Finsen “*in recognition of his contribution to the treatment of diseases*, *especially lupus vulgaris*, *with concentrated light radiation*, *whereby he has opened a new avenue for medical science*” (https://www.nobelprize.org/prizes/medicine/1903/summary/). However, the subsequent application of light radiation in medicine has been hampered by two major obstacles: the lack of controlled exposure systems and the lack of consistency of the proposed mechanisms of action. In the 1960s, the introduction of laser technologies opened a completely new phase, leading to the discovery of the biological effects of laser light low (LLL) doses [1]. Today, a huge body of evidence is available on LLL and photobiomodulation (PBM) in different *in vitro* and *in vivo* models, and a nomenclature consensus conference defined PBM as “*a form of light therapy that utilizes non-ionizing forms of light sources*, *including lasers*, *LEDs*” (light-emitting diodes) “*and broadband light*, *in the visible and infrared spectrum*” [2]. Other key concepts of PBM include “*nonthermal process involving endogenous chromophores eliciting photophysical* (*i.e.*, *linear and nonlinear*) *and photochemical events at various biological scales* (*…*) *resulting in beneficial therapeutic outcomes* (*…*) *promoting of wound healing and tissue regeneration*” [2–4]. In this context, the pioneer studies the Tiina Karu [5] has been the first to investigate the PBM effects [6], focusing on red and near-infra red light and their action on the mitochondria electron transport chain and on the unit IV cytochrome c oxidase.

Although the effects of PBM-based therapies are supported by a vast body of experimental evidence describing the effects of light stimulation on cell viability, cell proliferation, and tissue healing, suggesting that mitochondria and the cell membrane act as a support for molecular photoreceptors [7], the results of the various studies differ significantly. Many studies, for example, have shown that PBM stimulates wound healing by reducing the inflammatory response, improving angiogenesis, fibroblast proliferation, and collagen production, while others describe its inefficacy or even its inhibitory effects [8].

The standardization of PBM as treatment for tissue repair and regeneration has also been further hampered by a persistent lack of understanding of the cellular and molecular mechanisms underlying its action [4, 9], indeed most PBM studies lack controlled light delivery devices, standardized test procedures, validated multiple readouts, and *in vitro* models of pathological conditions.

To contribute to the standardization of PBM exposure protocols, and to clarify the underlying mechanisms of action, in this study we systematically explored the impact of different light wavelengths delivered by LEDs to fibroblasts, one of the main cell systems of interest for skin care, regeneration and repair, and the most suitable target because of their constant exposure to natural and artificial light. Because mitochondrial chromophores for red and near-infrared light have historically been considered those chiefly responsible for the effects of PBM [7], we used the mitochondrial membrane potential (MMP) measured by MitoTracker fluorescent dye staining as the major screening readout in our study, which involved six emitters at different wavelengths (440, 525, 645, 660, 780, and 900 nm). The selected emitter was then tested for its ability to prevent H_2_O_2_-induced oxidative stress in terms of mitochondrial integrity, reactive oxygen species (ROS) production, and cell death, as well as for its ability to modulate the frequency of the light wave.

## 2. Materials and Methods

### 2.1. LED Emitting Modules

The LED emitting module was prepared by Fremslife company (Genova, Italy). The experiment was designed to include a number of interchangeable fixtures, each holding a different wavelength LED. The optical power emitted by different LEDs differs widely, even between those with the same dimensions and produced by the same manufacturer.

**Figure 1:**
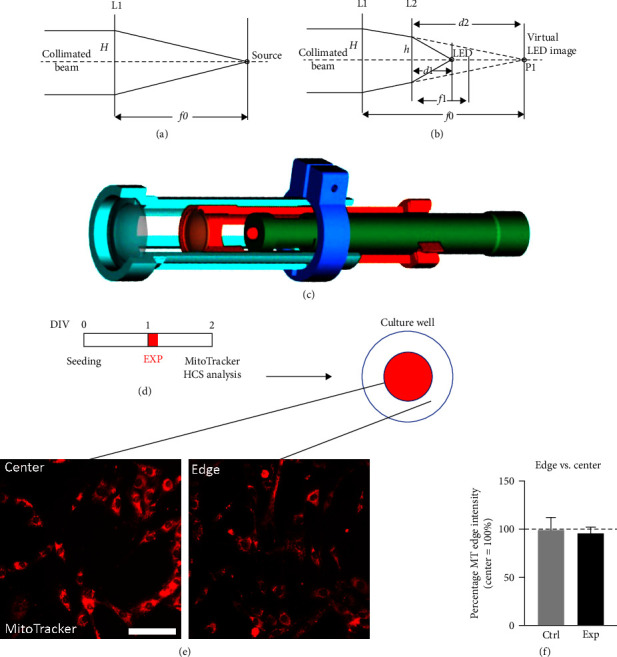
LED device and irradiation characterization. (a) Simple collimator. (b) Lens arrangement inside the collimator. (c) Cross section of the LED emitter. (d) Exposure protocol: 24 hr after seeding, cells were exposed to the red 645 nm light for 20 s. Exposure was performed in the center of the well only. After 24 hr, cells were stained with the MitoTracker fluorescent dye and fixed. (e) Representative pictures of cells stained with MitoTracker at the center of the well, where cells were directly exposed to the light, and at the edge of the well, where cells were not exposed to the light. Scale bar: 50 *µ*m. (f) The graph shows the relative MitoTracker intensity, and results are expressed as the percentage of the cells directly exposed to light at the center of the well (100%; white bar and dotted line); *n* = 3. Statistical analysis. Data are presented as average values ± SEM. Student's *t*-test was used to compare the MitoTracker intensity in the centre of the well (directly exposed to light) to the edge of each well (not exposed to light).

The selected diodes with a nominal continuous wave (CW) power ranging from 2.4 to 45 mW over radiation angles ranging from 15° to 25° were focused using an optical bank (two lenses) to give a spot of light with the same dimensions as the culture well at a fixed distance, and the supplied current was adjusted to give specific irradiance value (as indicated in Table 1). The factor that concerns us is the irradiance value; therefore, the ideal solution would have been to mount each diode in a collimator to give a parallel beam slightly larger in diameter than the target culture well, making the distance from the illuminator to the cell not relevant. For a point source, the simple solution is to locate it at the focus of a converging lens (Figure 1(a)), producing a collimated beam of the same diameter as the lens. The luminous flux reaching the lens, however, would be only a small part of the total flux emitted, greatly reducing its efficiency. The LEDs selected were all focalized by an integrated lens to produce a beam diverging at an angle of between 15° and 25°, using an arrangement of lenses as shown in Figure 1(b) (where the LED is schematized as a point source emitting over a finite solid angle). Lens L2 forms an image of the source at point P1, which is then collimated by lens L1. Different solid angles of emission can be accommodated by varying the position of lens L2 and the distance d1 from L2 to the source, using the same type (focal length and diameter) of lenses L1 and L2.

We compensated for the vast difference in optical output between the different LEDs by adjusting the diode current while measuring the output using a Thor Labs PM100D power meter adjusted to the appropriate wavelength, so that the resulting peak irradiance at the target had the prescribed value of 0.01 mW/cm^2^ (CW emission). The emission in this experiment was pulsed with a duty cycle of 1% for a mean irradiance of 0.001 mW/cm^2^ (Table 1). A cross section of the emitter has been included in Figure 1(c).

The individual current values were stored in the application software, and the individual emitter plugs were coded to allow the software to select the appropriate current, thus avoiding any operator bias.

### 2.2. Cell Culture

For the *in vitro* studies, we used the BJ human skin fibroblast (FBJ) cell line purchased from the American Type Culture Collection (ATCC® CRL-2522™, Manassas, VA, USA). Cells were cultured in Minimum Essential Medium (MEM, Gibco, Waltham, MA, USA) containing 5.5 mM D-glucose, supplemented with 10% heat-inactivated fetal bovine serum (FBS, Thermo Fisher Scientific, Waltham, MA, USA), 1% penicillin/streptomycin (100 U ml^−1^/100 *µ*g ml^−1^; Thermo Fisher Scientific), in a humidified incubator of 5% CO_2_ at 37°C. When 70%–80% confluency was reached, cells were passaged by trypsinization and subcultured in 25 cm^2^ flasks. For the LED exposure treatment, the FBS was removed from the culture medium. Cells were seeded con coverslips (10 mm diameter) placed in 24 well plates for epifluorescence imaging or directly cell-culture treated 96 well plate (Thermo Fisher Scientific), at a density of 10 × 10^3^ cells/cm^2^.

### 2.3. Light Exposure

**Figure 2:**
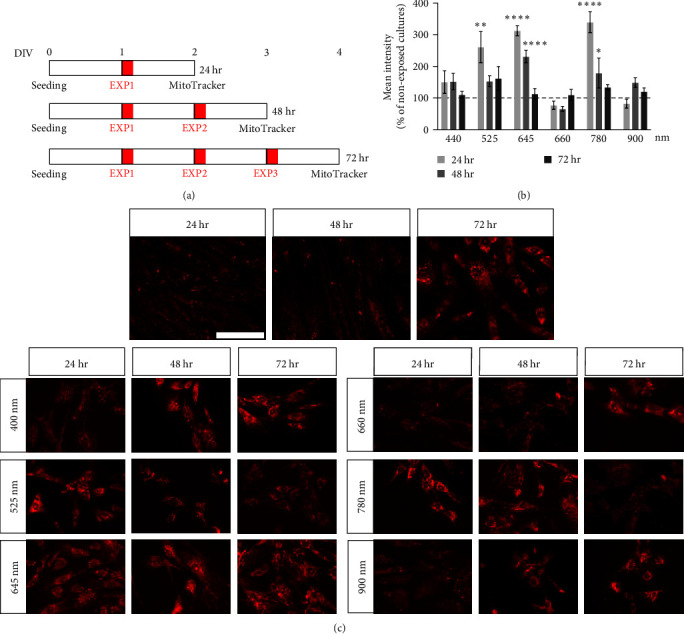
Effect of the different wavelengths on the mitochondrial membrane potential of fibroblasts. (a) Exposure protocol: cells were exposed for 20 s, either once, twice, or three times. Multiple exposures were performed at 24 hr intervals. Twenty-four hours after the final exposure, cells were stained with the MitoTracker fluorescent dye and fixed. Cultures not exposed to light were processed for the same time, and used as control groups for each protocol. (b) The graph shows the relative MitoTracker intensity, measured for each wavelength exposure (440, 525, 645, 660, 780, and 900 nm) and each exposure protocol (EXP1, EXP2, and EXP3). Results are expressed as a percentage of the nonexposed group (100%; horizontal dotted line) for each time point; *n* = 9 (nonexposed), 6 (exposed). (c) Representative images of cells exposed to different LEDs (440, 525, 645, 660, 780, and 900 nm) using the different protocols: EXP1 (24 hr), EXP2 (48 hr), and EXP3 (72 hr). Nonexposed control groups (Ctrl) are included in the panel. Scale bar: 50 *µ*m. Statistical analysis. Data are presented as average values ± SEM. One-way ANOVA followed by Dunnett's postdoc was used to compare groups in the same exposure protocol. Asterisks represent statistically significant differences between the indicated group and its corresponding nonexposed control ( ^*∗*^*P* < 0.05;  ^*∗∗*^*P* < 0.01;  ^*∗∗∗∗*^*P* < 0.0001).

LED exposures at the different wavelengths (440, 525, 645, 660, 780, and 900 nm) were performed for 20 s in all experiments. Three different protocols involving one, two, or three exposures at 24 hr intervals were used. Twenty-four hours after the final exposure, cells were stained with MitoTracker and fixed for image acquisition and analysis, as described in the following sections. A nonexposed group was used as a control culture (Figure 2(a)). Each experimental group was seeded on a separate plate, and the cells seeded at the extremity of each plate to avoid any exposure to reflected light. The plate was also covered with a sheet of dark paper with a single hole over the well with the same diameter as the well itself, to avoid the light accidentally hitting the nontarget wells. All cells were cultured and manipulated in the dark throughout the entire culture process, from seeding to fixation. Each well was exposed individually and because the exposure time was short (20 s) and each plate contained a single experimental group, the delay time between the different replicates has no impact on cell cultures and readout, as established by preliminary experiments.

**Figure 3:**
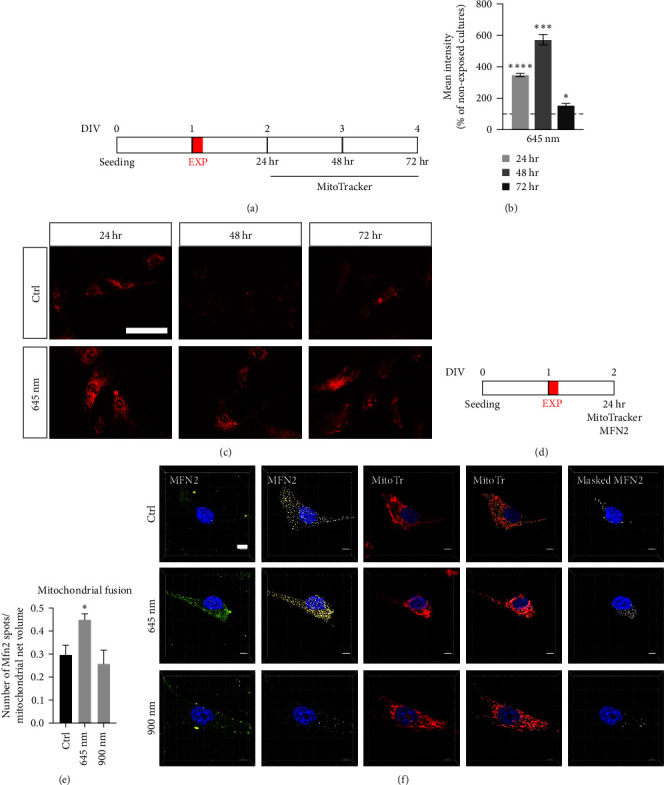
Mitochondrial analysis on fibroblasts exposed to 645 nm LED light. (a) Exposure protocol: using the red-emitting LED (645 nm), we performed a single 20 s exposure to analyze the potential mitochondrial changes after 24, 48, and 72 hr. At each time point, cells were stained with MitoTracker dye, fixed, and processed for analysis. (b) The graph shows the relative MitoTracker intensity, measured at each time point after exposure. Results are expressed as percentage of the nonexposed group (100%; horizontal dotted line) for each time point; *n* = 3. (c) Representative images of the nonexposed (Ctrl) and exposed cells (645 nm) 24, 48, and 72 hr after the exposure. Bar: 50 *µ*m. (d) Exposure protocol: to analyze mitochondrial fusion, we performed a single 20 s. exposure using two different LED light sources (645 and 900 nm). After 24 hr, cells were stained with MitoTracker, fixed, processed for immunocytochemistry for MFN2 (Mitofusin 2) marker identification, and processed for analysis. (e) Graph shows the ratio between the number of MFN2 spots and the total mitochondrial net volume per single cell, identified by the MitoTracker staining; *n* = 10. (f) Representative images of a cultured fibroblast, nonexposed (Ctrl) or exposed to the LED light (645 or 900 nm). Images were acquired by confocal microscopy as z-stacks and analyzed by IMARIS software. MitoTracker staining was initially used to create a mask to measure and isolate the mitochondrial net volume, then the “spot detection” algorithm was used to identify and count all MFN2 spots inside the identified volume. Scale bar: 5 *µ*m. Statistical analysis. Data are presented as average values ± SEM. One-way ANOVA followed by Dunnett's postdoc was used to compare groups in the same exposure protocol. Asterisks represent statistically significant differences between the indicated group and its corresponding nonexposed control ( ^*∗*^*P* < 0.05;  ^*∗∗*^*P* < 0.01;  ^*∗∗∗*^*P* < 0.001;  ^*∗∗∗∗*^*P* < 0.0001).

In another set of experiments, a single 20 s exposure was performed with the 645 nm wavelength, and the cells analyzed using the MitoTracker readout after 24, 48, and 72 hr (Figure 3(a)). To test the mitochondrial fusion using Mitofusin (MFN) immunostaining combined with MitoTracker, a single exposure was performed using 645 or 900 nm emitting devices, and the cultures analyzed after 24 hr (Figure 3(d)).

**Figure 4:**
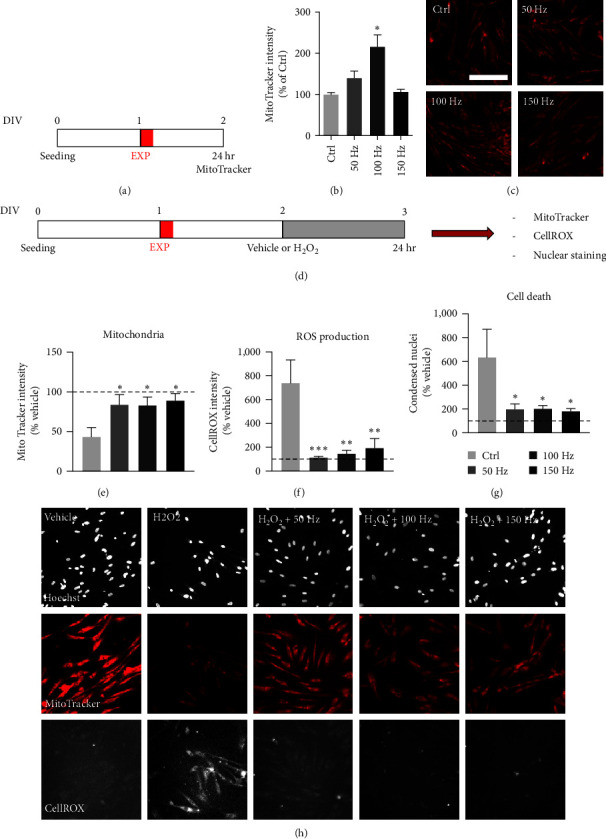
Effect of three different frequencies of the 645 nm LED light as a pre-exposure treatment on H_2_O_2_-induced oxidative stress. (a) Exposure protocol: to analyze the effect of the frequency, we performed a single 20 s. exposure using the 645 nm LED light at three different frequencies (50, 100, and 150 Hz) on fibroblast cultures. After 24 hr, cells were stained with MitoTracker, fixed, and processed for analysis by cell-based HCS. (b) The graph shows the relative MitoTracker intensity. Results are expressed as a percentage of the nonexposed group (100%; horizontal dotted line); *n* = 4. (c) Representative images of the nonexposed (Ctrl) and exposed cells. Scale bar: 100 *µ*m. (d) Exposure protocol: to analyze the possible protective effect against oxidative stress, we exposed the fibroblast cultures for 20 s, inducing oxidative stress through the H_2_O_2_ (40 *µ*M) treatment after 24 hr. After a further 24 hr, cells were stained with MitoTracker and CellROX dyes, fixed, stained with hoechst, and processed for the analysis by cell-based HCS. (e–g) Graphs show the effect on mitochondrial potential as the relative intensity of the MitoTracker staining (e), on ROS production as the relative intensity of CellROX staining (f), and cell death as relative percentage of condensed nuclei (g); *n* = 4. All the data are expressed as a percentage of the vehicle group not treated with H_2_O_2_ or exposed to the LED light (100%), whereas the control group treated with H_2_O_2_ but not exposed to the LED light is indicated by the white bar. (h) Representative HCS images of cells not treated with H_2_O_2_ or exposed to the LED light (vehicle); treated with H_2_O_2_ but not exposed to the LED light, and treated with H_2_O_2_ and exposed to the LED light at the three different frequencies (50, 100 and 150 Hz). Scale bar: 50 *µ*m. Statistical analysis. Data are presented as average values ± SEM. One-way ANOVA followed by Dunnett's postdoc was used to compare groups. Asterisks represent statistically significant differences between the indicated group and the nonexposed control (b) or the H_2_O_2_-treated/non-exposed control (e–g) ( ^*∗*^*P* < 0.05;  ^*∗∗*^*P* < 0.01;  ^*∗∗∗*^*P* < 0.001;  ^*∗∗∗∗*^*P* < 0.0001).

The following experiments were then performed on FBJ cultures to investigate the dynamics of the described effect on mitochondria and the effect of light frequency modulation, always using 20 s of exposure by the indicated LED light sources. For these experiments, cells were seeded on 96 well plates and analyzed by cell-based HCS, giving confirmation of the data obtained by conventional analysis while removing operator bias and permitting analysis of the entire culture. The frequency of the 645 nm light source was modulated from the 100 Hz used for all experimental sets to a lower (50 Hz) and a higher (150 Hz) frequency (Figure 4(a)).

Finally, to investigate any potential protective effect, we used the H_2_O_2_ treatment previously tested at different concentrations (Result S1 and Figure S2). Twenty-four hours after light exposure, cells were treated with H_2_O_2_ 40 *µ*M or vehicle (PBS) for 24 hr, stained with MitoTracker and CellROX, and fixed for image acquisition and analysis (Figure 4(d)).

### 2.4. Vital Staining of Mitochondria and Immunocytochemistry

As indicated in the different protocols, depending on the considered readout, cells were stained using MitoTracker™ Orange CMTMRos (Thermo Fisher Scientific) to visualize and analyze the mitochondrial net, and using CellROX (Thermo Fisher Scientific) to quantify ROS production.

MitoTracker was used at a concentration of 100 nM, whereas CellROX was used at a concentration of 5 *µ*M, following the manufacturer's instructions. Cells were incubated for 30 min at 37°C in a 5% CO_2_ humidified atmosphere. Following the live staining, cultures were fixed with 4% paraformaldehyde in 0.1 M Sørensen phosphate buffer for 20 min at room temperature (RT). After rinsing in phosphate buffered saline (PBS) solution 1x, cells were incubated with the nuclear dye Hoechst 33,258 (1 *μ*g/ml in PBS, 0.3% Triton-X 100) for 20 min at RT. Cultures on glass coverslips were then mounted on microscopy slides in 0.1% glycerol/1,4-phenilendiamine (Sigma–Aldrich), whereas cells in a 96-well plate were stored in PBS for HCS analysis.

For the confocal image analysis, cells were processed to identify the mitochondrial fusion marker Mitofusin 2 (MFN2). Cultures were stained with MitoTracker then fixed as described. After rinsing in PBS 1x, cells were incubated with 1% Donkey Normal Serum (Sigma–Aldrich) in 0.3% PBS/Triton-X 100 (Merck, Darmstadt, Germany) blocking solution for 1 hr at room temperature, and then incubated overnight at 4°C with the primary antibody (anti-MFN2; D2D10 - Rabbit, 1 : 500; #9482 Cell Signaling Technology) diluted in 0.3% PBS/Triton-100. After rinsing in PBS (two times for 10 min), cells were incubated with fluorochrome-conjugated secondary antibody (Anti-Rabbit Alexa Fluor 488 conjugated; 1 : 500; Jackson Laboratories) diluted in 0.3% PBS/Triton-X 100 for 30 min at 37°C, together with Hoechst 33,258 nuclear dye.

### 2.5. Imaging Acquisition and Analysis

To analyze the glass coverslips, we used a conventional epifluorescence microscopy using a Nikon Eclipse E600 microscope equipped with the digital CCD camera Q Imaging Retiga-2000RV (Q Imaging, Surrey, BC, CA), 620 nm filter, and NIS-Elements AR 3.2 software. A region of interest (ROI) was created around each cell in correspondence with the cell perimeter, measuring the fluorescence intensity of the MitoTracker staining inside each ROI. An ROI was also created inside the nucleus identified by the Hoechst staining, used to subtract the background in the MitoTracker emission channel.

Cells were also analyzed by the cell-based HCS machine (Cell Insight XT, Thermo Scientific), which acquires and analyzes the images via the dedicated software (HCS Studio v 6.6.0), using the “Compartmental analysis” BioApplication and the 386 nm (blue), 549 nm (red), and 650 nm (far-red) filters. Based on the nuclear staining, the software recognizes each individual cell in the analyzed wells, automatically generating a circular ROI inside the nucleus to analyze the nuclear morphology, and a ring-like ROI around the nucleus to analyze the cytoplasmatic staining. Using three color channels in parallel, we analyzed: (i) cell death based on the nuclear morphology, considering small and highly fluorescent nuclei as condensed; (ii) MMP, quantifying the MitoTracker fluorescence intensity; and (iii) oxidative stress, quantifying the CellROX fluorescence intensity. The HCS technology allows the automatic analysis of all cells in all wells involved in each experiment (3,000–4,000 cells/well), avoiding the operator bias inherent in manual field selection and drastically increasing the reliability and robustness of the data produced.

For the confocal analysis of the mitochondrial fusion, images were acquired with a Nikon Ti-E fluorescence microscope, connected to an A1R confocal system (Nikon, Minato, Tokyo, Japan) consisting of a series of diode lasers with an output wavelength of 405 nm, an air-cooled argon-ion laser system with 488 nm output, and a yellow diode-pump solid-state laser system with a 561 nm wavelength output. Images were acquired using a 40x lens with 1,024 × 1,024 resolution, and all z-stacks were collected in compliance with the optical section separation (z-Interval) values suggested by the NIS-Elements AR 3.2 software (1 *μ*m). Confocal z-stacks were then processed by IMARIS software (v.9.7; Andor Technology Limited, Belfast, UK). MitoTracker staining was used to construct an isosurface-based volume of the entire mitochondrial net, using the “surface” algorithm. The mitochondrial volume was used to mask the MFN2 channel, to isolate only the puncta corresponding to mitochondrial fusion. The “spot” algorithm was used on the MFN2 masked channel to detect all spots inside the mitochondrial net, which were then counted to analyze the mitochondrial fusion, obtained by dividing the MFN2 spots by the net mitochondrial volume per cell analyzed.

For all the analysis, data obtained from exposed group have been compared to nonexposed control cultures.

### 2.6. Statistical Analysis

At least three technical and biological replicates were performed for each experiment. The data are expressed as mean ± SEM, and one-way ANOVA followed by Dunnett's post-test was used to analyze the statistical differences. GraphPad Prism Software (v.9) was used to analyze the data, and results were considered significant when the probability of their occurrence as a result of chance alone was less than 5% (*P* < 0.05).

## 3. Results

### 3.1. LED Exposure System and Experimental Set-Up

Figure 1(c) shows a cross section of one of the emitting modules, while the main features of the exposure conditions are summarized in Table 1.

Because a LED is not a point source, we initially confirmed that the biological effect of exposure was uniform in the wells by measuring the main study readout (MMP by MitoTracker staining) at the center and edges of the well (Figures 1(d) and 1(e)). The absence of variations in MitoTracker intensity (Figure 1(f)) on cells in different positions in the culture wells (center or edge) confirmed the homogeneous effect of light exposure throughout the entire well. The analysis was performed both on coverslips mounted on coverglass and on 96 well plates, for conventional epifluorescence microscopy coupled with fluorescence intensity analysis through NIS software, and for HCS analysis, respectively.

### 3.2. LEDs at Different Wavelengths Have Different Effects on Fibroblast Mitochondria

The effect of the red light (670 nm) produced by a laser source on fibroblast MMP was previously described by our group [10]. We therefore used a mitochondrial readout, based on MitoTracker staining, to set the photobiomodulation (PBM) experimental conditions, opting for a very short exposure protocol (20 s) and three exposure conditions (1, 2, or 3 times with a 24 hr interval between exposures). Cultures were stained 24 hr after the last exposure. Culture timing and treatment conditions are shown in Figure 2(a) and were used to screen the effect of the different LED wavelengths, that is, 440, 525, 645, 660, 780, and 900 nm.

Exposure to certain wavelengths caused an increase in MitoTracker staining intensity (Figure 2(b)). Using the single exposure protocol, certain wavelengths increased the mitochondrial potential (one-way ANOVA, *F* (6.38) = 17.98, *P* < 0.0001), particularly 525 nm (Dunnett's post-test, *P* = 0.0003), 645 nm (*P* < 0.0001), and 780 nm (*P* < 0.0001). The effect was also measurable after two exposures (one-way ANOVA, *F* (6.41) = 6.434, *P* < 0.0001), confirming the effect of the 645 nm (Dunnett's post-test, *P* = 0.0001) and 780 nm (*P* = 0.0310) wavelengths. Three different wavelengths did not affect the considered readout in any of the protocols used (440, 660, and 900 nm), with no effects detected for any of the wavelengths following three exposures (one-way ANOVA, *F* (6.41) = 1.627, *P* = 0.1642). Representative images of the FBJ cultures are shown in Figure 2(c).

The wavelengths affecting the considered mitochondrial readout showed no cumulative effect with subsequent exposures, with a peak of MitoTracker fluorescence 24 hr after the single light exposure. Using the red-emitting LED (645 nm), we then investigated the MMP 24, 48, or 72 hr after a single 20 s exposure (Figure 3(a)). The MitoTracker intensity was higher at all study time points compared to nonexposed controls (Student's *t*-test, 24 hr, *P* < 0.0001; 48 hr, *P* = 0.0002; 72 hr, *P* = 0.0255) (Figure 3(b)), with a peak at 48 hr postexposure, and a substantial decline at 72 hr. Representative micrographs are shown in Figure 3(c).

The increase in MitoTracker intensity suggested a marked and reproducible effect on the mitochondrial compartment; therefore, we selected the most efficient protocol (the single exposure) to test the effect of light exposure on mitochondrial dynamics, using one of the wavelengths which had previously produced a high increase in MitoTracker fluorescence (645 nm), and one which had produced no effect (900 nm) (Figure 3(d)). Cells were stained with MitoTracker, and following fixation, the MFN2 protein was detected by indirect immunocytochemistry, and the nuclei identified via Hoechst staining. Mitochondrial fusion was visualized by confocal microscopy z-stacks, then analyzed using IMARIS software. We initially created the MitoTracker isosurface on the red fluorescence, defining the volume of the mitochondrial net using the MitoTracker volume to mask the MFN2 green staining, and applying the “spot” algorithm to the masked channel (Figure 3(f)). The number of MFN2 spots compared to the total mitochondrial net volume was used as the mitochondrial fusion index. We confirmed that the 645 nm light increased mitochondrial fusion, compared to the nonexposed control (one-way ANOVA, *F* (2.27) = 5.400, *P* = 0.0106; Dunnett's post-test, *P* = 0.0365) (Figure 3(e)).

### 3.3. LED Pre-Exposure Protects Cells against Oxidative Stress

Results obtained under baseline conditions indicate that light exposure induces an increase in MMP, a mechanism widely recognized as protective in response to PBM treatments also [11]. Increased mitochondrial fusion has also been described as potentially protective because fragmentation of the mitochondrial net destabilizes the membrane, promoting oxidative stress and leading to cell death [12].

To test the hypothesis of a protective effect exerted by the LED emitted light, we selected the most efficient LED emitter (645 nm) to investigate the potentially protective effect of the single exposure protocol on oxidative stress injury, a type of cell damage which directly involves the mitochondria, and which contributes to many pathological events. Previous studies have suggested that the 100 Hz pulse frequency may play a specific role in the observed phenomena related to mitochondria potential and dynamics [13]: to confirm this hypothesis, we decided to perform new experiments at one higher (150 Hz) and one lower (50 Hz) frequency, using the same MitoTracker intensity readout of the 645 nm LED emitter (Figure 4(a)). Only the 100 Hz frequency 645 nm light was able to replicate the previous increase in MMP compared to nonexposed controls (one-way ANOVA, *F* (3.10) = 5.691, *P* = 0.0155; Dunnett's post-test, *P* = 0.0257) (Figure 4(b)). Representative micrographs are included in Figure 4(c).

We tested the potential of all three frequencies to protect cells against oxidative stress, using a light pre-exposition protocol followed by H_2_O_2_ (40 *µ*M) as a toxic stimulus under the experimental conditions established by preliminary experiments (Result S1 and Figure S2). We tested different concentrations of H_2_O_2_ (20, 100, and 500 *µ*M, for 24 hr), which showed no toxicity at 20 *µ*M and high toxicity at 100 *µ*M. We therefore chose an intermediate value of 40 *µ*M to induce a mild cytotoxic effect (around 50% cell death).

To test the effect of PBM on oxidative stress-induced toxicity, we exposed the cells for 20 s to the three different frequencies of the 645 nm LED emitter, whereas the H_2_O_2_ toxic stimulus was added to the cultures after 24 hr and maintained for 24 additional hours (Figure 4(d)). Three different readouts were measured by cell-based HCS: MMP (MitoTracker staining intensity), ROS production (CellROX staining intensity), and cell death (percentage of condensed nuclei, Hoechst nuclear staining).

All tested frequencies restored the mitochondrial potential which had been drastically decreased due to oxidative stress (white column, H_2_O_2_ treated cultures) (one-way ANOVA, *F* (3.26) = 4.021, *P* = 0.0178; Dunnett's post-test, 50 Hz, *P* = 0.0364; 100 Hz, *P* = 0.0421; 150 Hz, *P* = 0.0129) reaching the level of the vehicle treated group (dotted horizontal line) (Figure 4(e)). ROS production, which had increased due to H_2_O_2_ exposure, was restored to the level of the vehicle-treated cultures (one-way ANOVA, *F* (3.28) = 7.843, *P* = 0.0006; Dunnett's post-test, 50 Hz, *P* = 0.0007; 100 Hz, *P* = 0.0013; 150 Hz, *P* = 0.0030) (Figure 4(f)) as was the induced cell death (one-way ANOVA, *F* (3.10) = 4.310, *P* = 0.0340; Dunnett's post-test, 50 Hz, *P* = 0.0428; 100 Hz, *P* = 0.0324; 150 Hz, *P* = 0.0259) (Figure 4(g)). Representative images are shown in Figure 4(h).

We also tested a postexposure protocol, exposing the cultures to light 1 hr after H_2_O_2_ treatment (Result S3 and Figure S4). However, none of the tested frequencies induced any measurable effect on mitochondrial potential, ROS production or cell death, and showed high variability between the replicates.

## 4. Discussion

The main objective of this study was to provide experimental evidence of the possible applications of PBM in skin care. We therefore focused on a single exposure with a very short exposure time (20 s) and long-lasting effects on MMP (72 hr after exposure). To the best of our knowledge, no data on these exposure conditions are available.

We used a highly standardized LED light-emitting device directed at fibroblasts, the type of skin cell most involved in physiological and pathological skin responses, with mitochondria and oxidative stress as the main readouts.

LED light-emitting devices have been widely studied for skin care and regeneration, with the literature describing the effect as varying based on the characteristics of the light, that is, light intensity, exposure time, and wavelength [14]. The wavelength used in skin treatment depends on the required penetration into the skin and the intracellular target [15–17]; however, the literature reveals that PBM testing is carried out in a variety of experimental settings which often fail to accurately measure this variable.

We used a PBM device specifically designed for experimental use which allowed complete control of the emitted light (see Table 1 for details). Different wavelengths in the visible light spectrum were tested, including blue, green, red (440, 525, 645, 660) and far-red/infrared (780, 900 nm). Different exposure protocols (1, 2, or 3 exposures at 24 hr intervals) were also tested.

We used a mitochondrial readout related to the effect of light exposure on MMP (*ΔΨ*m) increase [18, 19], measured by the MitoTracker fluorescent dye staining. The cell platform was based on BJ human fibroblasts because these cells display a lower MMP heterogeneity than other cell types [7]. To correlate MMP and the role of mitochondria in cell coupling to stress, we also analyzed mitochondrial fusion, which is considered a protective strategy helping to maintain energy output [20]. Mitochondria are renowned red photon light photoreceptors, and a recent systematic review defined PBM parameters on mitochondrial bioenergetics in different cell types very well [21]. Most studies have shown positive cell viability data when exposed to wavelengths from 660 to 940 nm at irradiances in the range of 200–400 *μ*W/mm^2^, for times ranging from 10 to 60 s. The corresponding energy delivery runs from a minimum of 2 mJ/mm^2^ to a maximum of 12 mJ/mm^2^. Overall in the literature, the parameters used are not consistent (and are sometimes even not used at all).

With these shortcomings in mind, we used the same irradiance of 0.1 mW/cm^2^ for all frequencies, and the same exposure time of 20 s for the radiation, pulsed at a 1% duty cycle. The net result was exposure to an irradiance of 0.001 mW/cm^2^ for a total time of 0.2 s, with 20 *μ*J/mm^2^ (20 J/m^2^) of energy delivered in every case, that is, at least two orders of magnitude less than the amount employed in the preceding studies.

MMP is an indicator of mitochondrial activity, correlating with adenosine triphosphate (ATP) production, redox balance, signaling, and cell metabolism [22]. The change in mitochondrial state due to PBM is usually evaluated by indirect methods, such as 3-(4,5-dimethylthiazol-2-yl)-2,5-diphenyl-2H-tetrazolium bromide assay, nicotinamide adenine dinucleotide + hydrogen quantification [23], and ATP synthesis [24]. Time lapse analysis of the mitochondrial dynamic is another excellent method [25]; however, it may introduce a strong bias because the imaging process (usually involving laser light exposure) is itself considered a form of PBM.

Having considered the different methods, we decided to use MitoTracker as an indicator for membrane potential, based on staining intensity. MitoTracker Orange is a cationic fluorophore, molecules which accumulate electrophoretically in mitochondria in response to the highly negative MMP, caused by the difference in potential between the mitochondrial matrix, plasma membrane, and external medium [7]. A number of studies have shown that the fluorescence intensity of this dye is directly affected by the MMP (see e.g., Ahmad et al. [26]): in particular MitoTracker Orange forms a covalent bound with thiols on proteins through the chloromethyl group, allowing the retention of the staining [27] and subsequent fixation. This characteristic makes MitoTracker Orange very stable once it has entered the mitochondria, creating a clear picture of mitochondrial status.

We have described the reliability of this method in various studies, showing the ability of MitoTracker Orange staining to detect the MMP decrease due to different noxious stimuli (e.g., oxygen and glucose deprivation, glutamate excitotoxicity) in different cell types (e.g., primary neuronal cultures, neural stem cells), and showing this condition to be an early stage of cell death [28, 29].

In this study, we observed a transient increase in MMP in fibroblasts (at 24 hr) following exposure to 525, 645, and 780 nm wavelengths, whereas no changes were observed at 440, 900, and at 660 nm using a 100 Hz frequency. Notably, this effect persisted 24 hr after exposure, with a further increase at 48 hr and recovery at 72 hr (645 nm). This matches the increase in mitochondrial fusion as measured by 3D image analysis of the mitochondrial fusion protein MFN2, localized in the outer membrane.

The different effects of the different wavelengths are not surprising. Red (650 nm) and blue irradiation (398 nm) have shown different effects on cytochrome *c* oxidase activation in hepatocarcinoma cells, despite both wavelengths exerting a similar effect on cell viability and mitochondrial fusion [30]. Six hundred and sixty nanometers light increases ROS production, whereas 970 nm light exerts a moderate antioxidant activity in PMNs and keratinocytes [31].

Moreover, the use of pulsed versus continuous light is a variable that is considered a key parameter affecting the biological outcome. In fact, it has been described that IR pulsed light mediates the ROS processes responsible for the interaction between cells and the extracellular matrix *in vitro* [32], and is more effective than the continuous light *in vivo* to induce wound healing [33].

Oxidative stress is one of the main factors in the aging of different tissues, including skin [34], and is directly involved in the pathogenesis of different skin diseases and wound healing processes [35–37]. The effect on mitochondrial redox signaling and attenuation of oxidative stress by exposure to red light is also reported in several cell types [7]. However, all these studies analyzed the short-term effects, that is, MMP or other parameters related to oxidative stress, immediately or shortly after exposure cessation. For example, 670 nm light treatment will diminish oxidative stress and prevent downstream inflammatory mechanisms in retinal ganglionic cells analyzed 60 min after the final exposure [38], whereas 625 nm LED exposure for 0.5 or 1 hr produces an ROS scavenging effect in immortalized keratinocytes [39].

We then investigated whether the increase in MMP due to PBM offers long-lasting protection against ROS-induced damage, using hydrogen peroxide as an oxidative stress stimulus. Based on the results obtained on the MMP at 72 hr postexposure with single or multiple exposures, we selected a single exposure to perform a 24 hr pretreatment. This pre-exposure to PBM offered the cells complete protection against ROS-induced cell death after a further 24 hr exposure to a mild dose of H_2_O_2,_ which killed almost 50% of the treated cells in culture. This protective effect is mediated by an ROS production block, and by maintaining mitochondrial status in a physiological-like MMP. This protection appears to be directly linked to the PBM-dependent effects generated in the 24 hr after the exposure, with a 1 hr exposure following H_2_O_2_ treatment unable to mediate any protective effects.

The kinetics of light-dependent changes in nitric oxide (NO) production have been also indicated as one of the possible factors responsible for the antioxidant effect of PBM [40]. Different light wavelengths generate different responses in terms of NO production, and multiple exposures with different wavelengths, in series or simultaneously, may generate synergistic and/or interfering effects. In this case too, however, readouts were only considered minutes after PBM, leaving its long-term effects unexplored. Given that many studies have hypothesized a slight increase in ROS production following PBM due to the “activation” of the cell [41], this “preconditioning” may explain the long-term effects observed in this study, including the protection mediated by pre-exposure, indeed ROS are not only negative factors which lead to cell damage; but also play many physiological roles. Regulated oxidative stress is a physiological signaling system, regulating mitochondrial fission and autophagy, which optimizes mitochondrial clearing, and may also be the trigger for the pathways involved in cell protection [42]. We then speculated that PBM-induced ROS production at a low level may initially mediate a positive activation of the entire biochemical system in charge of redox homeostasis, together with an increase in MMP, which may protect mitochondria from oxidative damage.

As already postulated, there is a strictly ROS-dependent correlation between ROS and MMP. Studies show that short exposures (5, 10, or 20 s) of a mesenchymal adipose stem cell line to a 830 nm laser increases both MMP and ROS over the short term [25], corroborating the hypothesis of a direct link between the two phenomena. Despite the high variability of the protocols and *in vitro* systems used to test the effects of PBM, most studies appear to conclude that PBM leads to a short-term stimulation of ROS production. It is difficult to find clear demonstrations of these mechanisms, however, because they are dependent on the combination of physical light source parameters such as wavelength and exposure timing, and on specific cell type. It has been proven, for example, that a long exposure time of almost three minutes with a wavelength of 780 nm stimulates ROS production in a cell line of squamous carcinoma within the first hour postexposure, whereas it has no effect on a line of human neonatal dermal fibroblasts. In both cells, however, the exposure generated a peak of ATP production in the first 4–6 hr, which rapidly normalized within 12 hr [43]. Moreover, in a clinical study using three different wavelengths (660, 800, and 970 nm), first tested on different cell cultures in which they generated different effects on ROS production, use of a laser in a patient with hyperoxidative status led to a reduction in oxidative parameters [31]. This suggests that the activated ROS production due to PBM is actually an activation of the ROS-eliminating system, leading to a more efficient clearing of ROS and protection of the cell.

## 5. Conclusions

The innovative LED emitting device used in the present study, allowing complete control of the emitted light parameters, would set a new quality standard in the field of PBM, allowing a clear description of the parameters used. This would in turn lead to an increase in data reproducibility, improving the translational power of preclinical experiments.

The solidity of the data obtained is also due to the coupling of cell platforms and well-established readouts with the HCS technology, together with the investigation into the long-term effects of PBM. Indeed, we were able to establish for the first time that MMP modification by very short light exposure not only persists for at least 72 hr, but also generates a protective intracellular environment which blocks ROS production, saving the cell from ROS-induced death.

## Figures and Tables

**Table 1:** Irradiation parameters.

Wavelength (nm)	Color	Nominal peak power (mW)	Used LED (data sheets available on request)	Current value for a irradiance of 0.1 mW/cm^2^ (mA)
440	UV	19–23 mW	LLS-UV400	3.65
525	Blue	100 cd	G58A5111P	4
645	Red	11 mW	LED-645-03	5.90
660	Red	15 mW	LED-660N-03	10
780	FR/IR	45 mW	ELD-780-525	11
900	IR	48 mW/sr	B5B-900-8	26
The irradiance was adjusted using a ThorLabs PM100D power meter				
Main probe characteristics:				
Emitter to target distance			120 mm	
Spot diameter and area at target distance			15.5–754.4 mm^2^	
Pulse radiation frequency			50–100–150 Hz	
Duty cycle peak irradiance			1% 0.1 mW/cm^2^	
Mean irradiance			0.001 mW/cm^2^	
Irradiation time			20 s	
Specific energy delivered			0.02 mJ/cm^2^	

The following table summarizes all the irradiation parameters standardized and controlled by the LED emitter device used in this study.

## Data Availability

All the data used to support the findings of this study are available from the corresponding author upon request.

## Supplementary Materials

Supplementary Materials Result S1: set up of the H_2_O_2_ treatment conditions for the oxidative stress experiment. Figure S2: set up conditions for the H_2_O_2_ induced oxidative stress protocol. Result S3: the LED light postexposure does not protect cells from oxydative stress. Figure S4: the effect of three different frequencies of the 645 nm LED light as a postexposure treatment on the H_2_O_2_ induced oxidative stress.
