# Supplementary material for: Photobiomodulation at Defined Wavelengths Regulates Mitochondrial Membrane Potential and Redox Balance in Skin Fibroblasts
Source: Oxid Med Cell Longev. 2023 Aug 24;2023:7638223. doi: 10.1155/2023/7638223 (PMC10471456; doi:10.1155/2023/7638223)
Supplement: Supplementary Materials — Result S1: set up of the H2O2 treatment conditions for the oxidative stress experiment. Figure S2: set up conditions for the H2O2 induced oxidative stress protocol. Result S3: the LED light postexposure does not protect cells from oxydative stress. Figure S4: the effect of three different frequencies of the 645 nm LED light as a postexposure treatment on the H2O2 induced oxidative stress. [file 7638223.f1.pdf]

Supplementary results

Oxidative medicine and cellular longevity

Photobiomodulation at defined wavelengths regulates mitochondrial membrane potential and redox balance in skin fibroblasts

Vito Antonio Baldassarro,[1,2] Giuseppe Alastra,[1] Luca Lorenzini,[1,2] Luciana Giardino[1,2] and Laura Calzà[2,3,4]

[1] Department of Veterinary Medical Sciences, University of Bologna, Via Tolara di Sopra, 50, Ozzano dell'Emilia (Bologna) 40064, Italy. [2] Interdepartmental Centre for Industrial Research in Health Sciences and Technologies, University of Bologna, Via Tolara di Sopra, 41/E, Ozzano dell'Emilia (Bologna) 40064, Italy. [3] Department of Pharmacy and Biotechnology, University of Bologna, Via Belmeloro, 6, Bologna 40126, Italy. [4] IRET Fundation, Via Tolara di Sopra, 41/E, Ozzano dell'Emilia (Bologna) 40064, Italy.

Authors contacts: V.A.B. vito.baldassarro2@unibo.it; G.A. giuseppe.alastra2@unibo.it; L.L. luca.lorenzini8@unibo.it; L.G. luciana.giardino@unibo.it

Correspondence should be addressed to Laura Calzà; laura.calza@unibo.it

Result S1. Set up of the H_2O_2 treatment conditions for the oxidative stress experiment

On fibroblast cells, we used 24 hours incubation with different H_2O_2 concentrations (0, 20, 100 and 500 µM), according to the standard literature on this in vitro oxidative stress model, to test the optimal concentration for the light exposure test. The H_2O_2 was added in the culture media which was deprived of serum, to avoid the antioxidant effects of different serum component (Supplementary Figure S1A). Three different readouts were measured by cell-based HCS: mitochondria potential (MitoTracker staining intensity), ROS production (CellROX staining intensity) and cell death (percentage of condensed nuclei, Hoechst nuclear staining). However, since in the highest concentrations was not possible to measure the percentage of condensed nuclei due to the detachment of most of the cells, we used the cell number as a parameter correlated to cell viability, since we plated the same number of cells for all the experimental groups.

The H_2O_2 treatment induced modification in the mitochondria potential (One-Way ANOVA, $F(3,12) = 174.7$, $P < 0.0001$), with a reduction of the MitoTracker staining intensity at 100 µM (Dunnett's post-test, $P < 0.0001$) and 500 µM ($P < 0.0001$) (Supplementary Figure S1B). At the same concentrations, also the ROS production was affected by the oxidative stress induction (One-Way ANOVA, $F(3,12) = 6.479$, $P = 0.0074$), with a marked increase of the fluorescence intensity (Dunnett's post-test, 100 µM, $P = 0.0162$; 500 µM, $P = 0.0056$) (Supplementary Figure S1C). The number of cells at the end of the 24 hours H_2O_2 exposure was drastically affected (One-Way ANOVA, $F(3,12) = 24.29$, $P < 0.001$) with a strong reduction for the two highest concentrations (Dunnett's post-test, 100 µM, $P = 0.0003$; 500 µM, $P = 0.0002$) (Supplementary Figure S1D). Representative pictures are included in Supplementary Figure S1E.

Since the 100 and 500 µM concentrations induced a strong toxic effect resulting in the death and detachment of most of the cells, we used an intermediate concentration (40 µM) between the 20 µM (no effect) and the 100 µM (strong effect).

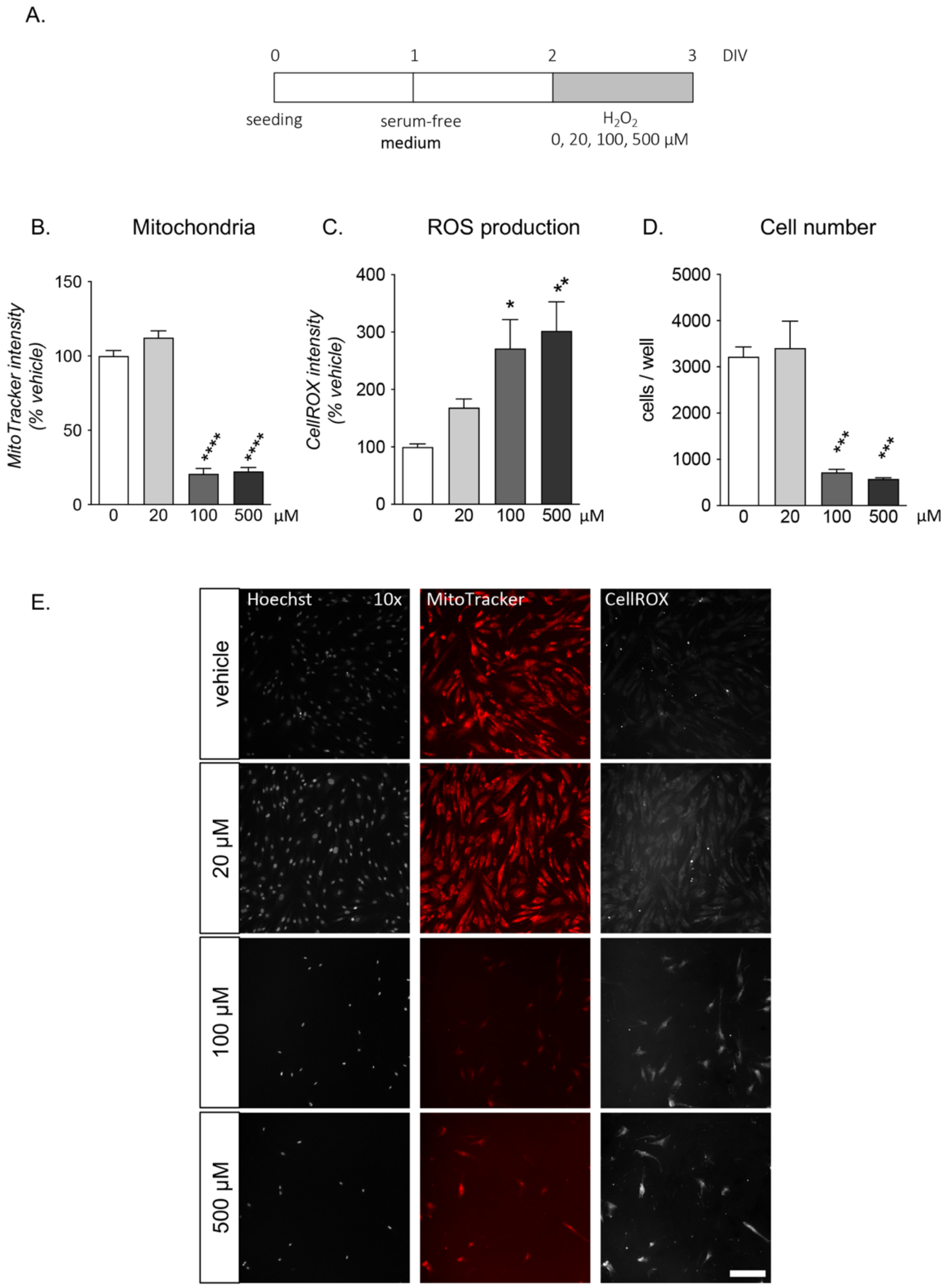

Supplementary Figure S1: Set up conditions for the H_2O_2 induced oxidative stress protocol. (A) Protocol: we used 24 hours incubation with different H_2O_2 concentrations (0, 20, 100 and 500 µM) to test the optimal condition for the light exposure test. The H_2O_2 was added in the culture media which was deprived of serum, to avoid the antioxidant effects of different serum component. (B - D) Graphs show the measure of the relative MitoTracker intensity (B; 0 µM = 100%), relative CellROX intensity (C; 0 µM = 100%) and the total number of cells per well (D). Statistical analysis: data are presented as average values ± SEM. One-Way ANOVA followed by Dunnett's post-doc. Asterisks represent statistically significant differences between the indicated group and the vehicle-treated (0 µM) group (* $P < 0.05$; ** $P < 0.01$; *** $P < 0.001$; **** $P < 0.0001$).

Result S2. The LED light post-exposure does not protect cells from oxidative stress.

We replicated the exposure experiment (see Figure 4 in the main text), which resulted protective from the H_2O_2 induced oxidative stress, but changing the timing of exposure to a post-exposure: cells were first treated with H_2O_2 and, after 1 hour, exposed to the LED red light (645 nm) at three different frequencies (50, 100 and 150 Hz). After 24 hours, cultures were than stained and fixed to analyze the selected readouts, i.e. mitochondria potential using MitoTracker intensity, ROS production using CellROX intensity, and cell death analyzing the nuclear morphology through Hoechst nuclear staining. Analysis were performer in HCS technology, analyzing the whole number of cells per each well and condition (Supplementary Figure S2A).

The post-exposure protocol showed no effect on the mitochondria potential compared to the group which was treated with H_2O_2 and not exposed to the light (Supplementary Figure S2B), as well as for CellROX intensity (Supplementary Figure S2C) and cell death (Supplementary Figure S2D). Representative images are included in Supplementary Figure S2E.

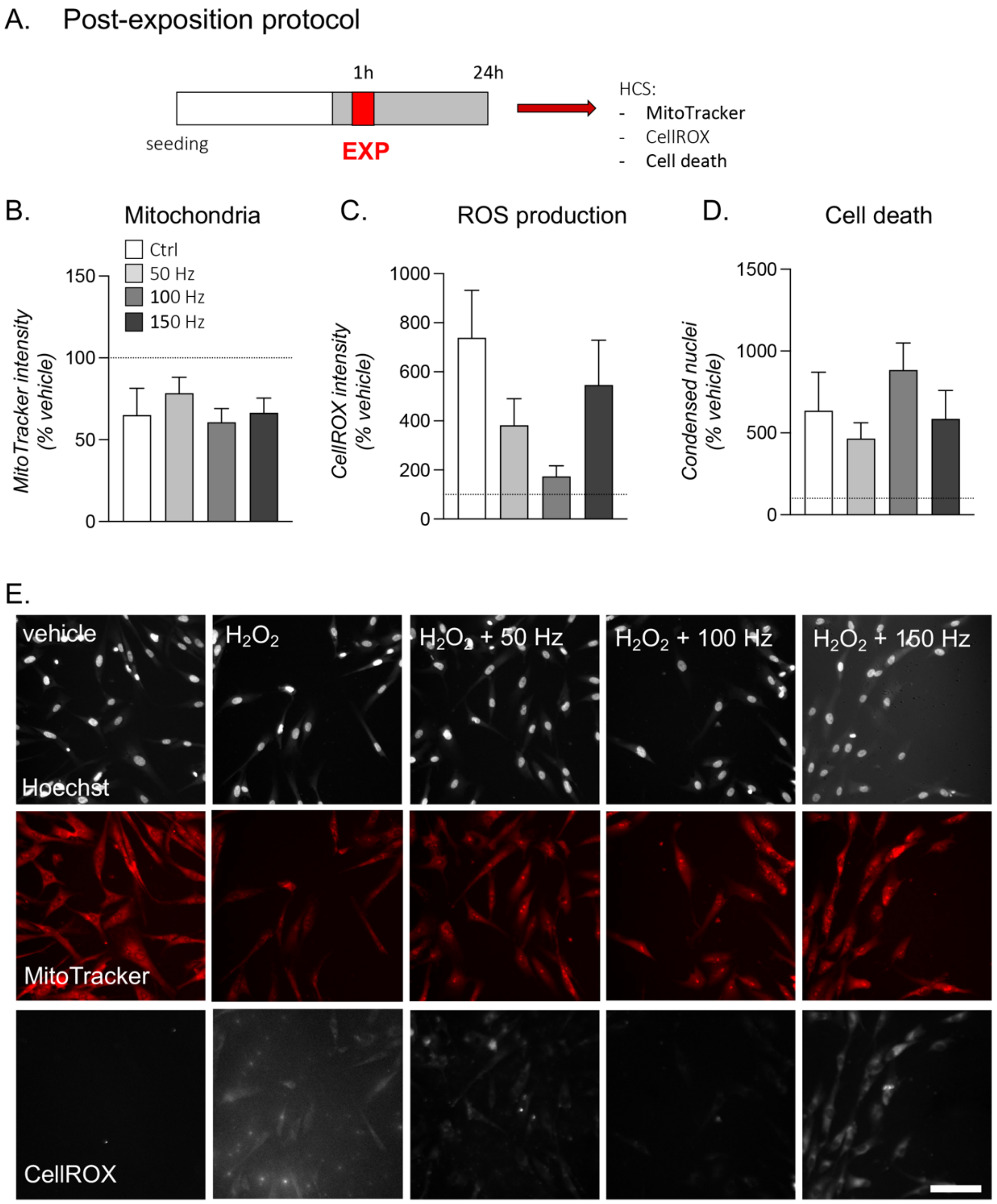

Supplementary Figure S2. The effect of three different frequencies of the 645 nm LED light as a post-exposure treatment on the H_2O_2 induced oxidative stress. (A) Exposure protocol: to analyze the possible protective effect on oxidative stress, we treated cells with H_2O_2 40 µM for 24 hours. One hour after the H_2O_2 induction we exposed the fibroblast cultures for 20 sec at the 645 nm LED light. At the end of the H_2O_2 treatment, cells were stained with MitoTracker and CellROX dies, fixed, stained with Hoechst and processed for the analysis by cell-based High Content Screening. B – D) Graphs show the effect on mitochondria potential as relative intensity of the MitoTracker staining (B), on ROS production as relative intensity of CellROX staining (C), and cell death as relative percentage of condensed nuclei (D). All the data are expressed as percentage of the vehicle group non-treated with H_2O_2 and non-exposed to the LED light (100%), while the control group treated with H_2O_2 and non-exposed to the LED light is indicated with the white bar. (E) Representative HCS images of cells non-treated with H_2O_2 and non-exposed to the LED light (vehicle), treated with H_2O_2 and non-exposed to the LED light, and treated with H_2O_2 and exposed to the LED light at the three different frequencies (50, 100, 150 Hz). Statistical analysis: data are presented as average values ± SEM. One-Way ANOVA followed by Dunnett's post-doc was used to compare groups.
